# Editorial: Introducing *Surgery Open Science*

**DOI:** 10.1016/j.sopen.2019.04.002

**Published:** 2019-04-30

**Authors:** Nicholas J. Zyromski

**Affiliations:** Editor-in-Chief, Surgery Open Science

Dear Colleagues:

We live in an exciting, dynamic time for science, medicine, and publication. Rapid technological and clinical advances are being disseminated more rapidly than ever before imagined. In this milieu, Elsevier is very pleased to bring you a new surgical journal—*Surgery Open Science*. This open-access journal is published as a sister journal to *Surgery*, one of the leading subscription journals in the field of surgery. The primary goal of *Surgery Open Science* is to deliver the *highest quality* open access surgical basic and clinical science content, with leading edge dynamic, transparent, and interactive access to process and data.

The open access movement has roots beginning in the 1960s; however, it really began to gain traction in early 21st century as internet availability spread widely. In brief, gold open access makes all of a journal's content available to anyone at any time. The journal's ability to curate their data is covered financially by article processing charges to funding agencies, institutions, or authors. The advantages of open access are numerous, and include immediate and universal access to content, exposure to a very broad audience, and publication on an electronic platform with unlimited space requirements.

The open access movement has been embraced particularly in the life science community which has used this platform in creative ways such as ongoing data accumulation, providing an open forum for dynamic discussion of research findings, and completely transparent access to the peer review process.

Over the past decade, open-access publication has grown rapidly. For example, in 2016, it is estimated that 15% of all medical scientific articles were published in open-access journals [1]. In September, 2018, Science Europe, an association of European research institutions and funding agencies proposed “Plan S.” This plan mandates that all research funded by public grants be published in compliant open access journals [2].

A significant challenge for the open access movement is quality control. Academic medical professionals are solicited seemingly daily by so-called “predatory” open access publications—disreputable journals focused primarily on financial gain. Development of high-quality open access platforms in the surgical space therefore represents a critical need. In this milieu, *Surgery Open Science* is extremely well-positioned, with full backing of the Elsevier publishing infrastructure and support of the existing Elsevier surgery journal family. Elsevier is supporting a *Surgery Open Science* Managing Editor to ensure that all manuscripts meet outlined technical specifications, monitor the reviewing process, and ensure each author receives an outstanding publication experience with their submission. *Surgery Open Science* uses the same production team as the prestigious Elsevier print surgical journals, including the latest high-tech proofing tools. The *Surgery Open Science* editorial team has recruited an experienced and enthusiastic editorial board to provide oversight to a competitive review process; all manuscripts undergo the same rigorous peer review process expected at subscription journals such as *Surgery*.

A fundamental goal of *Surgery Open Science* is to provide the highest quality science in the open access space. Our first issue underscores this commitment to quality. The top-quality articles in the first issue including review of circulating tumor cells by world class Johns Hopkins University scientists highlight the quality expected by the Editorial Board and supported completely by Elsevier.

We look forward to the journal's evolution, engaging with our readership as *Surgery Open Science* grows.
